# Single versus bilateral internal thoracic artery grafting in patients with impaired renal function

**DOI:** 10.1371/journal.pone.0297194

**Published:** 2024-02-14

**Authors:** Ariel Farkash, Amit Gordon, Rephael Mohr, Orr Sela, Dmitri Pevni, Tomer Ziv-Baran, Ayelet Grupper, Jonathan E. Kfir, Yanai Ben-Gal

**Affiliations:** 1 Department of Cardiothoracic Surgery, Tel Aviv Sourasky Medical Center and Faculty of Medicine, Tel Aviv University, Tel Aviv, Israel; 2 Department of Epidemiology and Preventive Medicine, School of Public Health, Faculty of Medicine, Tel Aviv University, Tel Aviv, Israel; 3 Department of Nephrology, Tel Aviv Sourasky Medical Center and Faculty of Medicine, Tel Aviv University, Tel Aviv, Israel; Ataturk University Faculty of Medicine, TURKEY

## Abstract

**Objective:**

The optimal strategy for surgical revascularization in patients with impaired renal function is inconclusive. We compared early and late outcomes between bilateral internal thoracic artery (BITA) and single ITA (SITA) grafting in patients with renal dysfunction.

**Methods:**

This is a retrospective analysis of all the patients with multivessel disease and impaired renal function (estimated glomerular filtration rate <60mL/min/1.73m^2^) who underwent isolated coronary artery bypass graft (CABG) in our center during 1996–2011, utilizing either BITA or SITA revascularization.

**Results:**

Of the 5301 patients with multivessel disease who underwent surgical revascularization during the study period, 391 were with impaired renal function: 212 (54.2%) underwent BITA, 179 (45.8%) underwent SITA. Patients who underwent BITA were less likely to have comorbidities. Statistically significant differences were not observed between the BITA and SITA groups in 30-day mortality (5.6% vs. 9.0%, p = 0.2) and in rates of early stroke, myocardial infarction, and sternal infection (4.5% vs. 6.1%, p = 0.467; 1.7% vs. 2.8%, p = 0.517; and 2.2% vs. 5.7%, p = 0.088, respectively). Long-term survival of the BITA group was better: median 8.36 vs. 4.14 years, p<0.001. In multivariable analysis, BITA revascularization was associated with decreased late mortality (HR = 0.704, 95% CI: 0.556–0.89, p = 0.003). In analysis of a matched cohort (134 pairs), early outcomes did not differ between the groups; however, in multivariable analysis, BITA revascularization was associated with decreased late mortality ‎‎(HR = 0.35 (95%CI 0.18–0.68), p = 0.002)‎.

**Conclusions:**

BITA revascularization did not impact early outcome in patients with CRF, but demonstrated a significant protective effect on long-term survival ‎in the unmatched and matched cohorts.

## Introduction

With the aging of the general population, reduced renal function has become an increasingly common comorbidity among persons who undergo surgical myocardial revascularization [1]. As chronic kidney disease (CKD) is known to be associated with poor outcome in coronary artery bypass graft (CABG) surgery [2, 3], CKD is included in the two most established surgical risk assessment tools (Society of Thoracic Surgeons score and EuroSCORE) [4, 5].

In the general population, bilateral internal thoracic artery (BITA) grafting for coronary revascularization has demonstrated improved long-term survival, compared to single ITA (SITA) revascularization [6–8]. Yet, little is known on the early and long-term outcomes of BITA revascularization in patients with CKD.

BITA revascularization is the preferred strategy in our center for most individuals with multivessel coronary artery disease (CAD), including those with pronounced comorbidities. This study investigated early and long-term outcomes of left-sided (left anterior descending (LAD) and left circumflex artery territory) revascularization with BITA vs. SITA in patients with renal dysfunction.

### Patients and methods

This study comprised all the patients with multivessel coronary disease who underwent isolated CABG in Tel-Aviv Medical Center during 1996–2011, deploying either both ITAs to the left side or only a single ITA to the LAD, with or without additional grafts (mostly saphenous vein grafts) to non-LAD lesions. Subjects’ data was collected during 2022. Authors had access to patients’ identifying information during data collection.

The Institutional Review Board of Tel Aviv Sourasky Medical Center approved the study. As the data were analyzed anonymously, the Institutional Review Board of Tel Aviv Sourasky Medical Center approved the waiver of consent.

For this study, we calculated estimated glomerular filtration rates (eGFR) of all our patients, using the CKD-Epidemiology Collaboration Creatinine Equation *(eGFRcr = 142 x min(Scr/κ*, *1)α x max(Scr/κ*, *1)-1*.*200 x 0*.*9938Age x 1*.*012 [if female])*, a four-variable formula [9]. An eGFR value of <60mL/min/1.73m2 was the cut off for CKD and for inclusion in the current analysis [10]. Demographic data, clinical characteristics, early outcomes (death, stroke, myocardial infarction (MI), revision for bleeding, and deep sternal wound infection (DSWI)), and late survival were compared between patients who underwent BITA and SITA grafting.

In our center, the BITA revascularization strategy is employed also for high-risk patients [11]. We engage a more cautious approach towards the BITA strategy in patients with an increased risk for DSWI, such as those with chronic obstructive pulmonary disease (COPD), women with obesity or diabetes mellitus (DM), and elderly patients (aged >80 years). Still, the final decision to perform BITA or SITA grafting is at each surgeon’s discretion (all the surgeons use both techniques). ITA grafts are skeletonized during harvest; additional technical aspects were detailed in previous publications of our group [12, 13].

EuroSCORE clinical data standards were used to analyze patient data [14]. Early mortality was defined as death within the index revascularization or during the first postoperative month. A peri-procedural MI was defined as the appearance of new Q-waves or ST-segment elevation of more than 2mm on electrocardiograph, accompanied by a creatine phosphokinase-myocardial band greater than 50 mU/mL, with or without a regional wall motion abnormality [15, 16]. A cerebrovascular accident was defined as a new long-lasting neurological impairment, established by computed tomography evidence. DSWI was defined as a sternal or peri-sternal infection (with clinical and laboratory evidence) requiring an open surgical intervention. An emergent operation was defined as an operation performed within 24h of catheterization or in patients with evident pre-operative acute or evolving MI, pulmonary edema, or cardiogenic shock. A critical preoperative state was defined as preoperative ventricular tachycardia or fibrillation, aborted sudden death, preoperative ventilation, or pre-operative insertion of an intra-aortic balloon pump counterpulsation (IABP).

Early outcomes (during the index hospitalization) were drawn from patients’ charts and medical records, discharge letters, and the department database. The Israeli National Registry database was examined to obtain full and comprehensive information regarding late mortality. We also compared outcomes by stratifying the data by performance during 2000 and earlier, and after 2000. This analysis aimed to minimize bias related to the change of surgical team, the technical learning curve and improved technology or pharmacological therapy.

### Statistical analysis

Categorical variables were described as frequencies and percentages. Continuous variables were evaluated for normal distribution using histograms, and reported as means and standard deviations, or medians and interquartile ranges. The chi-square test and Fisher’s exact test were used to compare categorical variables between the two surgical strategies, and the independent samples t-test and Mann-Whitney tests were applied to compare continuous variables. Follow-up duration was determined by the reverse censoring method. Kaplan Meier curve analysis was used to describe survival during the follow-up period and to report median survival time. The log-rank test was used to compare survival between the two surgical strategies. Multivariable Cox regression was applied to evaluate the association between mortality and surgical strategy, while controlling for possible known confounders. The regression contained four blocks. In the first block, surgical strategy was forced into the regression. In the second block, age, gender, and pre-operative parameters were considered as potential variables for inclusion in the model. In the third block, operative parameters were also considered potential variables for inclusion in the model. In the second and third blocks, all relevant variables were entered into the model and then the backward method was applied (the Wald test was used and p>0.1 was the criterion for removal). Finally, surgical era was forced into the regression in the fourth block. The two groups were matched according to the probability of a patient undergoing CABG using BITA. The probability (propensity score) was calculated using a logistic regression model. The following parameters were used to calculate the propensity score: sex, age, insulin-dependent DM, COPD, the use of an IABP, baseline eGFR, emergency procedures, redo procedures, left main disease, unstable angina pectoris, and neurologic dysfunction. Fuzzy matching without replacement was performed. An absolute difference (matching tolerance / caliper) in the propensity score of up to 5% (on a scale of 0 to 100%) was considered acceptable for matching [12, 17–20]. Standardized differences were calculated to compare the two groups before and after matching. A standardized difference of <0.1 was considered a negligible difference and a difference between 0.1 and 0.2 was considered a small difference [21, 22]. The matched groups were compared using the McNamar test for the categorical variables, and the paired t-test and Wilcoxon test for the continuous variables. Stratified Cox regression by pairs was used to compare survival between the matched groups. The regression contained four blocks. In the first block, surgical strategy was forced into the regression. In the second block, age, sex, and pre-operative parameters were considered as potential variables for inclusion in the model. In the third block, operative parameters were also considered potential variables for inclusion in the model. In the second and third blocks, all the relevant variables were entered into the model and then the backward method was applied (the Wald test was used and p>0.1 was the criterion for removal). Finally, surgical era was forced into the regression in the fourth block. All statistical tests were two-sided and p<0.05 was considered statistically significant. Statistical analysis was performed with SPSS statistical software (IBM SPSS Statistics for Windows, version 27, IBM Corp., Armonk, NY, USA, 2020). Visualization was performed with R (version 4.2.1, R-Foundation Statistical-Computing, Austria, 2022) and "prodlim" package (Product-Limit Estimation for Censored Event History Analysis) from November 13^th^ 2019.

## Results

**Fig 1:**
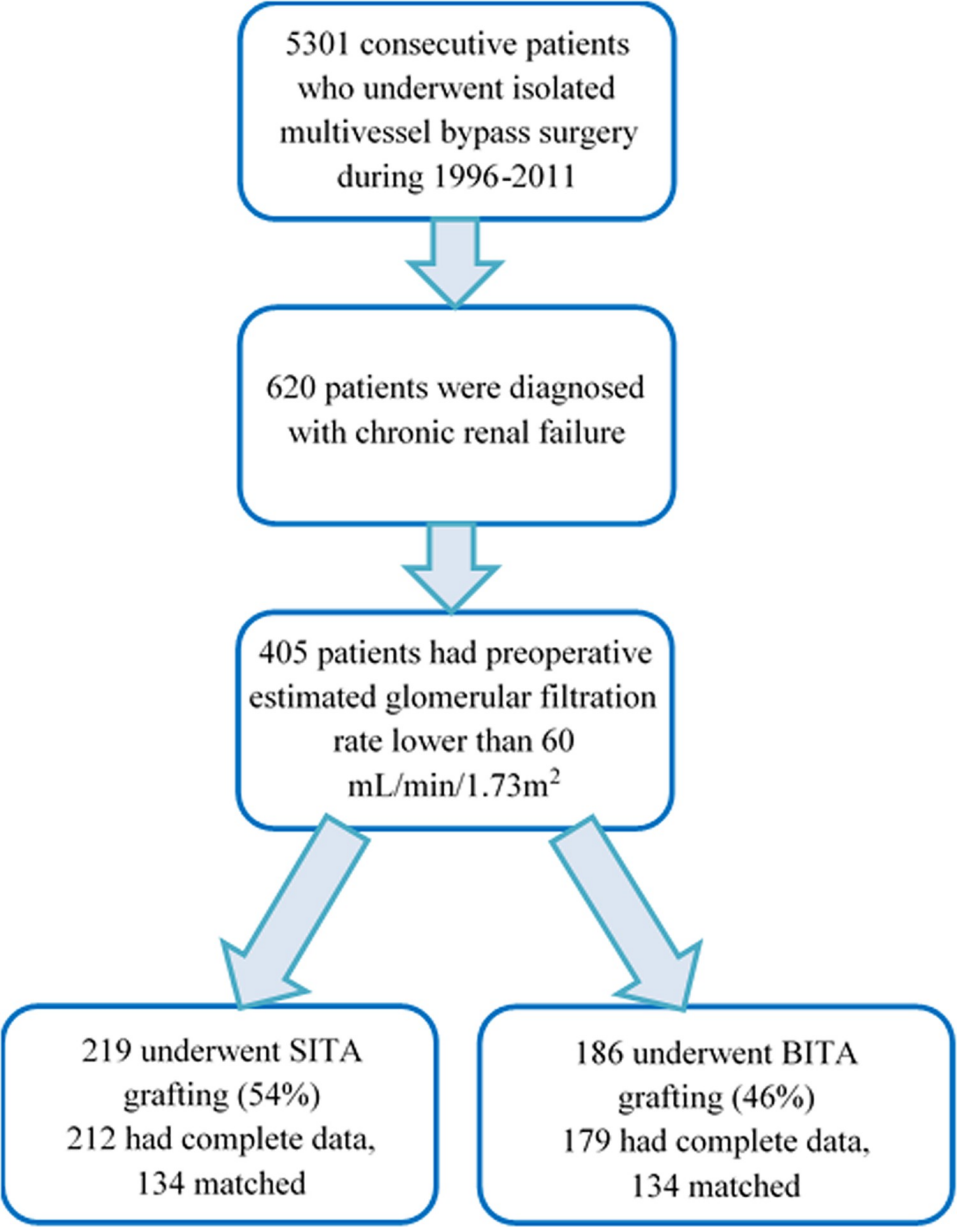
Flow diagram of the study.

Between January 1996 and December 2011, 5301 consecutive patients underwent isolated CABG surgery at our institution. We identified 405 patients who presented with renal dysfunction (eGFR <60mL/min/1.73m2) during their index hospitalization for revascularization. After excluding 14 patients without available data, the final cohort comprised 391 patients, of whom 212 (54.2%) underwent BITA grafting and the remaining 179 (45.8%) underwent SITA grafting (**Fig 1**). Saphenous vein grafts comprised most of the additional non-ITA grafts in both groups. We isolated 134 propensity-matched pairs of patients for an additional sub-analysis of both groups (**Fig 1**).

### Baseline characteristics of the unmatched cohort

Preoperative patient characteristics differed significantly between the BITA and the SITA groups. In the BITA compared to the SITA group, the mean age was younger and a smaller proportion was female. Patients in the BITA group were less likely to have a lower eGFR, a higher EuroSCORE, co-morbidities such as insulin-dependent DM, COPD, and unstable angina; and to have undergone preoperative IABP insertion, redo surgery, or emergency procedures.

Vein grafts, radial artery, and grafts to the right system were less commonly used in the BITA than the SITA group, and the proportion of off-pump procedures was lower. A higher proportion of patients in the BITA than SITA group received a right gastroepiploic artery as an additional arterial graft (**Table 1**).

**Table 1:** Preoperative and intraoperative characteristics of patients with chronic renal failure who underwent coronary artery bypass graft, according to matched and unmatched cohorts.

	All								
		SITA	BITA	p value	Standardized Mean Difference	SITA	BITA	p value	Standardized Mean Difference
	n = 391	n = 212 (54.2%)	n = 179 (45.8%)			n = 134	n = 134		
**Male**	321(82.1%)	164(76.9%)	159(88.8%)	0.001	0.31	118(88.1%)	114(85.1%)	0.572	0.088
**Age (years), mean (SD)**	71.72(9.8)	72.87(9.76)	70.36(9.69)	0.008	0.258	72.1(10.24)	71.62(8.89)	0.382	0.05
**Age ≥70**	262(67%)	149(70.3%)	113(63.1%)	0.134	0.152	94(70.1%)	90(67.2%)	0.652	0.064
**Baseline creatinine (IQR)**	1.8(1.6–2.3)	1.9(1.6–2.5)	1.8(1.53–2.1)	0.038	0.281	1.8(1.59–2.3)	1.8(1.5–2.17)	0.396	0.161
**Baseline eGFR, mean (SD)**	33.64(12.77)	31.56(13.6)	36.1(11.26)	<0.001	0.364	34.14(13.23)	35.24(11.12)	0.392	0.09
**IDDM**	41(10.5%)	29(13.7%)	12(6.7%)	0.025	0.232	15(11.2%)	12(9%)	0.69	0.074
**DM**	205(52.4%)	115(54.2%)	90(50.3%)	0.434	0.079	71(53%)	70(52.2%)	>0.999	0.015
**DM_EOD**	175(44.8%)	94(44.3%)	81(45.3%)	0.857	0.018	60(44.8%)	63(47%)	0.813	0.045
**COPD**	46(11.8%)	34(16%)	12(6.7%)	0.004	0.297	17(12.7%)	12(9%)	0.383	0.12
**CHF**	160(40.9%)	84(39.6%)	76(42.5%)	0.57	0.058	50(37.3%)	59(44%)	0.306	0.137
**Old MI**	207(52.9%)	114(53.8%)	93(52%)	0.72	0.036	75(56%)	70(52.2%)	0.635	0.075
**Acute MI**	84(21.5%)	48(22.6%)	36(20.1%)	0.544	0.062	23(17.2%)	25(18.7%)	0.875	0.039
**Unstable angina pectoris**	248(63.4%)	146(68.9%)	102(57%)	0.015	0.248	84(62.7%)	84(62.7%)	>0.999	0
**EF<30%**	50(12.8%)	31(14.6%)	19(10.6%)	0.237	0.121	15(11.2%)	15(11.2%)	>0.999	0
**IABP**	38(9.7%)	27(12.7%)	11(6.1%)	0.028	0.227	8(6%)	9(6.7%)	>0.999	0.031
**Critical preoperative state** *	38(9.7%)	22(10.4%)	16(8.9%)	0.632	0.049	6(6%)	12(9%)	0.503	0.18
**Emergent operation** **	78(19.9%)	56(26.4%)	22(12.3%)	<0.001	0.363	20(14.9%)	22(16.4%)	0.868	0.041
**Redo**	14(3.6%)	13(6.1%)	1(0.6%)	0.003	0.314	1(0.7%)	1(0.7%)	>0.999	0
**PVD**	157(40.2%)	89(42%)	68(38%)	0.422	0.082	58(43.3%)	55(41%)	0.81	0.045
**NOVS**	315(80.6%)	169(79.7%)	146(81.6%)	0.646	0.047	108(80.6%)	111(82.8%)	0.761	0.058
**Left main disease**	120(30.7%)	74(34.9%)	46(25.7%)	0.049	0.201	46(34.3%)	41(30.6%)	0.568	0.08
**Prior PCI**	72(18.4%)	41(19.3%)	31(17.3%)	0.607	0.052	24(17.9%)	24(17.9%)	>0.999	0
**EuroSCORE, median (IQR)**	10(7–13)	11(8–14)	9(6–11)	<0.001	0.639	10(7–12)	9(7–11.25)	0.062	0.21
**Logistic**	0.1655(0.0754–0.3371)	0.2425(0.0963–0.4641)	0.1108(0.059–0.2117)	<0.001	0.673	0.1658(0.0748–0.3087)	0.1303(0.0674–0.2513)	0.089	0.237
**Bypass≥3**	261(66.8%)	135(63.7%)	126(70.4%)	0.16	0.143	90(67.2%)	91(67.9%)	>0.999	0.016
**Sequential anastomoses number**	174(44.2%)	98(46.2%)	75(41.9%)	0.391	0.087	66(49.3%)	52(38.8%)	0.092	0.211
**SVG**	221(56.5%)	162(76.4%)	59(33%)	<0.001	0.97	106(79.1%)	47(35.1%)	<0.001	0.993
**GEA**	37(9.5%)	9(4.2%)	28(15.6%)	<0.001	0.388	4(3%)	20(14.9%)	0.002	0.428
**Radial**	45(11.5%)	43(20.3%)	2(1.1%)	<0.001	0.652	27(20.1%)	1(0.7%)	<0.001	0.669
**Right system revascular-ization**	273(69.8%)	164(77.4%)	109(60.9%)	<0.001	0.362	106(79.1%)	81(60.4%)	0.002	0.415
**OPCAB**	126(32.2%)	74(34.9%)	52(29.1%)	0.217	0.126	46(34.3%)	45(33.6%)	>0.999	0.016
**Operated after the year 2000**	248(63.4%)	167(78.8%)	81(45.3%)	<0.001	0.736	107(79.9%)	62(46.3%)	<0.001	0.742

BITA: bilateral internal thoracic artery grafting, CHF: congestive heart failure, COPD: chronic obstructive pulmonary disease, CRF: chronic renal failure, DM: diabetes mellitus, DM_EOD: diabetes mellitus with end organ damage, eGFR: estimated glomerular filtration rate, EF: ejection fraction, GEA: gastroepiploic artery, IABP: intra-aortic balloon pump counterpulsation; IDDM: insulin-dependent diabetes mellitus, IQR: interquartile ratio, MI: myocardial infarction, NOVS: number of vessel disease, OPCAB: off-pump coronary artery bypass, PCI: percutaneous coronary intervention, PVD: peripheral vascular disease, Redo: redo operation, SMD: standard mean difference, SITA: single internal thoracic artery grafting, SVG: saphenous vein graft.

*Defined as preoperative ventricular tachycardia or fibrillation, aborted sudden death, preoperative ventilation, or pre-operative insertion of an IABP.

**Defined as an operation performed within 24h of catheterization or in patients with evident pre-operative acute or evolving MI, pulmonary edema, or cardiogenic shock

### Early outcomes including (30-day) mortality, the unmatched analysis

Between patients who underwent BITA and SITA grafting, significant differences were not observed in early mortality (5.6% vs. 9.0%, p = 0.2), DSWI (2.2% vs. 5.7%, p = 0.08), stroke events (4.5% vs. 6.1%, p = 0.46), and perioperative MI (1.7% vs. 2.8%, p = 0.51). The occurrence of acute kidney injury (AKI, defined as a creatinine after/before ratio ≥1.5, or an increase by ≥0.3 during the index hospitalization) did not differ between the groups. However, the mean postoperative eGFR remained higher following BITA than SITA (**Table 2**). In multivariable analysis, male sex and critical preoperative state were associated with higher prevalence of postoperative AKI; the odds ratios were 1.79 (95% confidence interval [CI] 1.01–3.18), p = 0.047 and 2.4 (95%CI 1.15–5.00), p = 0.02, respectively. In a multivariable analysis, OPCAB surgery was not associated with patient survival, neither in the BITA and SITA groups, nor in the entire cohort. However, in multivariable analysis, OPCAB surgery was associated with lower prevalence of AKI in the SITA group only (OR = 0.5, 95% CI = 0.27–0.92), p = 0.026.

**Table 2:** Early outcomes of patients with chronic renal failure who underwent coronary artery bypass graft, according to matched and unmatched cohorts.

	All	Unmatched cohort n (%)			Matched cohort n(%)		
		SITA	BITA	p value	SITA	BITA	p value
	n = 391	n = 212	n = 179		n = 134	n = 134	
**Early mortality**	29(7.4%)	19(9%)	10(5.6%)	0.204	7(5.2%)	9(6.7%)	0.804
**Deep infection**	16(4.1%)	12(5.7%)	4(2.2%)	0.088	7(5.2%)	4(3%)	0.549
**Post CVA**	21(5.4%)	13(6.1%)	8(4.5%)	0.467	9(6.7%)	6(4.5%)	0.607
**Perioperative MI**	9(2.3%)	6(2.8%)	3(1.7%)	0.517	3(2.2%)	2(1.5%)	>0.999
**Revision for Bleeding**	14(3.6%)	11(5.2%)	3(1.7%)	0.063	4(3%)	2(1.5%)	0.687
**AKI**	154(39.5%)	83(39.3%)	71(39.7%)	0.947	54(40.6%)	52(38.8%)	0.791
**Postoperative eGFR** (mL/min/1.73m^2^) **after, mean (SD)**	30.85(14.05)	28.89(14.12)	33.15(13.66)	0.003	30.72(14.58)	32.41(13.75)	0.327

AKI: acute kidney injury, creatinine after/before ratio ≥1.5 or increased by ≥0.3, BITA: bilateral internal thoracic artery grafting, CVA: cerebrovascular accident, eGFR: estimated glomerular filtration rate, MI: myocardial infarction, SD: standard deviation, SITA: single internal thoracic artery grafting.

### Late outcomes of the unmatched cohort

**Fig 2:**
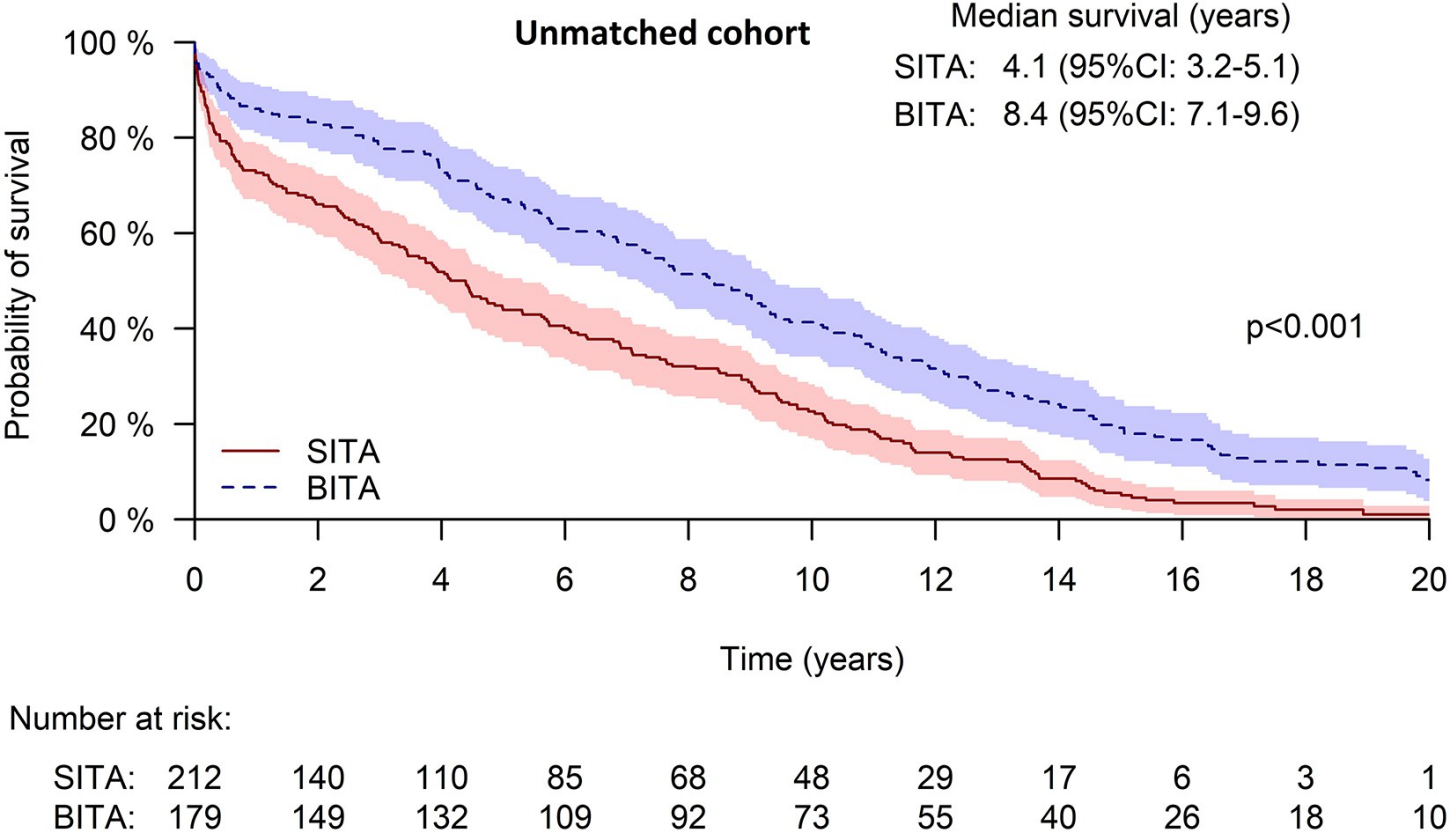
Kaplan-Meier curve of survival for the unmatched cohort.

While the follow-up time reached 20 years, the median survival for the whole cohort was 5.87 years. For the BITA and SITA groups, the median survival times were 8.36 and 4.14 years, respectively p<0.001; ten-year survival rates were 41.3+/-3.7% and 22.6+/-2.9%, respectively (**Fig 2**).

In multivariable analysis, BITA revascularization was associated with better late survival (adjusted hazard ratio [HR] 0.704, 95%CI: 0.556–0.89), p = 0.003; while older age, DM with end organ damage, COPD, peripheral vascular disease, left main disease, and lower preoperative eGFR were associated with decreased long-term survival after surgery (HR 4.07, 95%CI: 2.26–6.31, p<0.001 for eGFR <15mL/min /1.73m2).

### Outcomes according to the matched cohort

The 134 pairs of patients created in the propensity-matched analysis were well-matched in their pre-operative characteristics (**Table 1**). The BITA and SITA groups did not differ significantly in early (30-day) mortality (6.7% vs. 5.2%, p>0.80) and in the occurrences of early postoperative DSWI, peri-operative MI, postoperative stroke, and postoperative AKI (**Table 2**). In multivariable analysis, BITA revascularization was found to be associated with fewer AKI occurrences; the HR was 0.55 (95%CI 0.35–0.87), p = 0.011. DM was associated with a higher prevalence of AKI; the HR was 1.97 (95%CI 1.09–3.57), p = 0.025.

**Fig 3:**
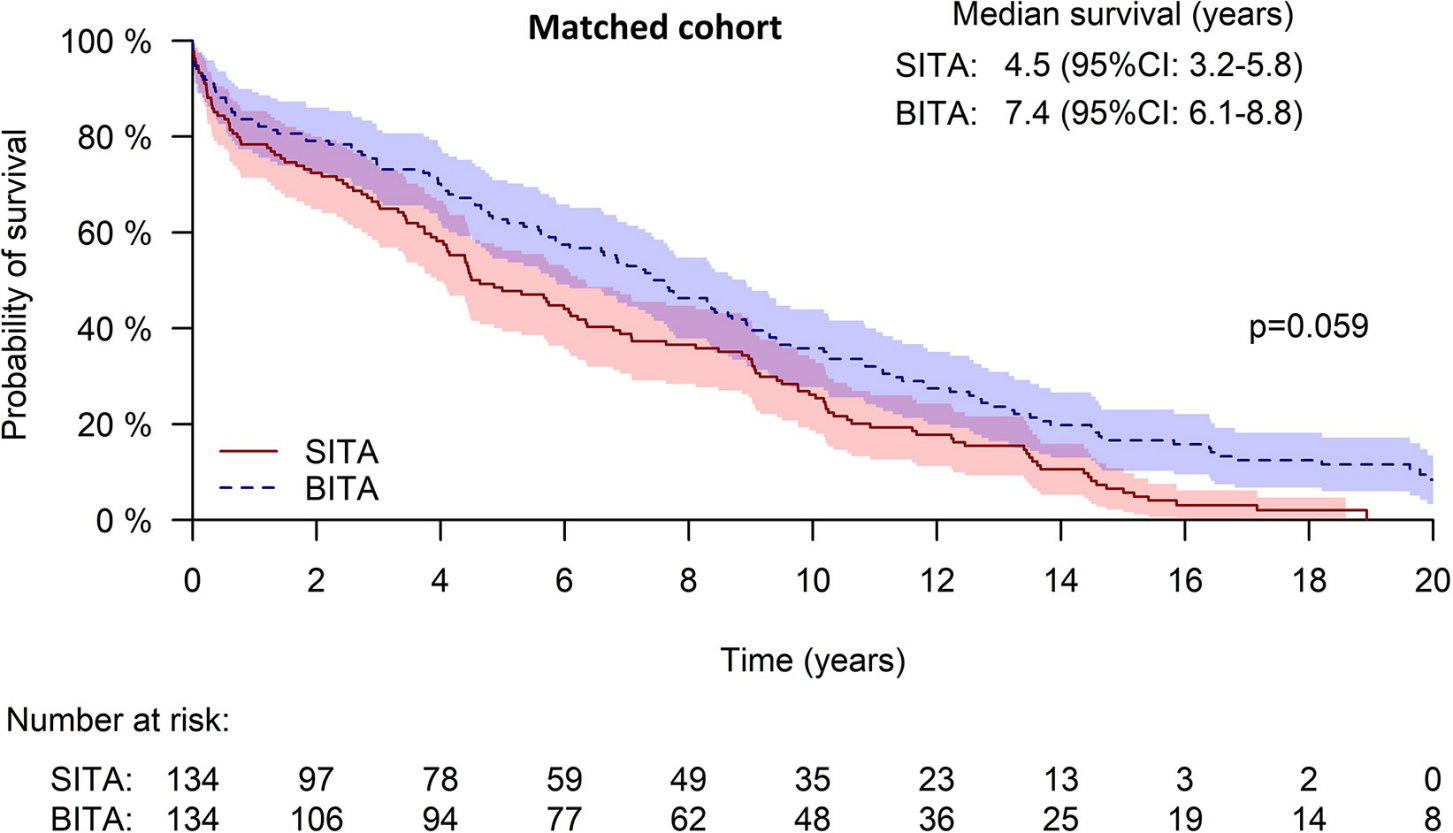
Kaplan-Meier curve of survival for the matched cohort.

For the matched groups, Kaplan Meier curves showed a trend towards better long-term survival for the BITA group (**Fig 3**). The 20-year survival rates were 35.8+/-4.1% and 26.1+/-3.8% in the BITA and SITA groups, respectively (p = 0.059). In multivariable analysis (to better control possible confounders in the matched cohort), BITA revascularization was associated with improved survival; the HR was 0.347 (95%CI 0.178–0.678), p = 0.002 **(Table 3)**.

**Table 3:** Multivariable analysis for 20-year overall mortality of the matched ‎cohort.

Predictor	HR (95% CI)	p value
**BITA**	0.347 (0.178–0.678)	0.002
**NIDDM**	1.747 (0.991–3.082)	0.054
**COPD**	3.787 (1.284–11.175)	0.016
**SVG**	2.299 (1.011–2.229)	0.047
**Radial artery**	3.649 (1.109–12.009)	0.033

BITA: bilateral internal thoracic artery grafting, CI: confidence interval, COPD: ‎chronic obstructive pulmonary disease, HR: hazard ratio, NIDDM: non-‎insulin-dependent diabetes mellitus, SVG: Saphenous vein graft.‎

Similar results were found after truncating the results at 5 and 10 years, (**Table 4**). In a univariate analysis for 5- and 10-year survival of the matched cohort, BITA ‎revascularization was not found to have a significant protective effect (p = 0.104 and ‎p = 0.088, respectively).‎ In a multivariable analysis for the matched cohort, truncated at 5 and 10 years, ‎BITA was associated with improved survival: HR 0.511 (95%CI 0.291–0.897), p = 0.019 ‎and 0.522 (95%CI 0.315–0.865), p = 0.012, respectively. This is comparable to the HR ‎of the total 20- year follow up: 0.347 (95%CI 0.178–0.678), p = 0.002.‎

**Table 4:** Univariate and multivariable analysis for the association between the ‎surgical strategy (BITA vs. SITA) and patient survival.

		Univariate		Multivariable*	
		HR (95%CI)	p value	HR (95%CI)	p value
**5-years**	**Unmatched**	0.481 (0.352–0.658)	<0.001	0.641 (0.454–0.906)	0.012
	**Matched**	0.714 (0.476–1.072)	0.104	0.511 (0.291–0.897)	0.019
**10-years**	**Unmatched**	0.566 (0.443–0.724)	<0.001	0.729 (0.554–0.958)	0.023
	**Matched**	0.732 (0.512–1.047)	0.088	0.522 (0.315–0.865)	0.012
**20-years**	**Unmatched**	0.561 (0.454–0.693)	<0.001	0.704 (0.556–0.890)	0.003
	**Matched**	0.718 (0.509–1.012)	0.059	0.347 (0.178–0.678)	0.002

BITA: bilateral internal thoracic artery grafting, CI: confidence interval, HR: hazard ratio, SITA: single internal thoracic artery grafting.

*Multivariable analysis—adjusted for age and sex.

We performed a sub-analyses, to evaluate associations of renal dysfunction severity at presentation and revascularization strategy ‎with survival. The entire cohort was stratified according to eGFR ‎‎<30mL/min/1.73m2 (141 patients) and eGFR > = 30mL/min/1.73m2 (250 patients). For both groups, BITA revascularization ‎showed a survival advantage. Among patients with lower ‎eGFR, the 10-year survival rates were 21.6+/-5.8% and 13.3+/-3.6%, p = 0.018, ‎for the BITA and SITA groups, respectively. Among patients with higher eGFR, the ‎‎10-year survival rates were 49.2+/-4.4% and 29.5+/-4.1%, p<0.001, for the respective groups.

Finally, we performed additional sub-analyses of patients with eGFR<15, eGFR = 15–30, eGFR = 30–45, and eGFR = 45–60 mL/min/1.73m2, as the reference group. The HRs for mortality were 4.07 (CI95% 2.625–6.31) p<0.001, 2.018 (CI95% 1.445–2.819) p<0.001 and 1.545 (CI95% 1.147–2.081) p = 0.004, respectively (S1 Fig). Data regarding the patient with ESRD and hemodialysis treatment were not available at the time of data collection.

## Discussion

CKD is a prominently growing epidemiologic concern [23, 24], and is highly associated with cardiovascular diseases, most particularly with CAD [3]. The latter is itself one of the leading causes of deaths in persons with CKD [24]. Renal dysfunction poses higher risks of uremia-related conditions such as anemia and calcium-phosphorus metabolism disturbances, of atherosclerotic burden, and of co-morbidities such as hypertension and DM [2, 3, 25].

Few studies [26–28] have evaluated the optimal treatment approach in the context of CAD with renal dysfunction. Mostly, they demonstrated a positive survival effect for CABG compared to percutaneous coronary interventions and medical therapy alone in patients with severe CAD eligible for surgical revascularization.

Despite the above, CKD itself, even with mildly impaired renal function, has been shown to poorly affect CABG outcomes. In their review of almost 484,000 individuals after CABG, with various degrees of renal dysfunction, Cooper et al. [4] found an inverse relation between short-term outcomes and the degree of renal-dysfunction. The same deleterious effects of renal impairment on midterm survival and major adverse cardiovascular events were found among patients in the SWEDEHEART registry [29]. In that study, ITA to the LAD artery attenuated adverse effects compared to a strategy without ITA. Another report [30] identified impaired creatinine clearance as an independent predictor for reduced two-year survival.

A number of targeted studies [7, 31–36] reported better late outcomes (mostly only one decade after surgery) among patients with multivessel CAD who underwent surgical revascularization deploying BITA rather than SITA.

The observed attenuated advantage of multi-arterial revascularization is generally attributed to the late attrition of vein grafts occurring predominantly five to ten years after surgery [37–39]. This accelerated atherosclerosis, associated with the use of saphenous vein grafts, is probably more pronounced in high-risk patients with renal dysfunction [40]. Yet, as these patients seldom received more than a single ITA, data are limited regarding the survival benefit of BITA in patients with renal dysfunction.

Kinoshita et al. [41] followed patients with CKD who underwent CABG for a median 5 years, and reported lower all-cause and cardiac-related late mortality for BITA vs SITA grafting across all CKD stages. Notably, for almost all the patients, BITA grafts were deployed using the "*in situ*" configuration, and the second ITA was not used exclusively to the left circumflex artery. In their meta-analysis, Tam et al. [42] found no survival benefit of BITA at 3.7 years among patients on dialysis who underwent surgical revascularization.The main finding of our study is the better long-term (up to 20 years) survival of patients with impaired renal function who underwent skeletonized left-sided BITA revascularization, compared to those who underwent CABG with SITA, mostly with additional vein grafts. The better survival for BITA versus SITA was apparent in both the unmatched and propensity matched cohorts (the latter showed only a strong trend). Moreover, in multivariable analysis, BITA revascularization was again associated with reduced mortality, even among these multi-morbid, high-risk patients. We speculate that this could be explained by a shift in the positive prognostic effect of the multi-arterial grafting, to an earlier phase than in the general population of patients without an augmented atherosclerotic process.

This single-center observational retrospective analysis has a number of limitations. First, for many patients, data were not available of complete follow-up of post-discharge renal function, and of major adverse cardiovascular events, as well as cardiac related mortality, after the index hospitalization. Such data could have revealed hidden or even more pronounced outcome differences of one revascularization strategy over the other. Due to the unavailability of data regarding dialysis performance and timing in the small group of patients with low eGFR, dialysis dependability was not included in the analysis. In addition, the exact angiographic characteristics (lesion type, severity, and syntax score) were unavailable. Importantly, the surgical strategy, i.e. BITA or SITA revascularization, was determined by each surgeon during the procedure, and stringent guidelines did not dictate the inclination of one strategy over the other. Accordingly, treatment allocation bias cannot be ruled out. Finally, intraoperative parameters, like third graft preference or on vs. off pump procedure, were not included in the matching process. This precluded further conclusions regarding specific revascularization techniques.

In conclusion, this study showed comparable early outcomes for BITA and SITA revascularization in patients with impaired renal function. However, the BITA grafting strategy demonstrated a trend towards long-term survival benefit, and a clear advantage in multivariable analysis. Further studies are needed to determine the exact role of BITA revascularization in these high-risk patients.

## Supporting information

S1 Checklist STROBE statement—checklist of items that should be included in reports of observational studies—PONE-D-23-11606. (DOCX)

S1 Fig Kaplan-Meier survival curves for each estimated glomerular ‎filtration rate group: eGFR≤15, 15>eGFR≤30, 30<eGFR≤45, and eGFR>45 mL/min/1.73m2. (DOCX)

S2 Fig Standardized mean difference plot before and after matching. (DOCX)

S1 Table Univariate Cox regression of additional factors that significantly affected 20-year survival of patients with chronic renal failure who underwent bilateral internal thoracic artery grafting. (DOCX)
