# Supplementary material for: Single versus bilateral internal thoracic artery grafting in patients with impaired renal function
Source: PLoS One. 2024 Feb 14;19(2):e0297194. doi: 10.1371/journal.pone.0297194 (PMC10866522; doi:10.1371/journal.pone.0297194)
Supplement: S1 Fig — (DOCX) [file pone.0297194.s002.docx]

**S1 Figure 1**

As presented in the below figure, the survival benefit for those who underwent BITA ‎revascularization was statistically significant only in the eGFR groups of 15-30 and ‎‎30-45 mL/min/1.73m2. A possible explanation is that both the low (less than 15 ‎mL/min/1.73m2) and high (45-60 mL/min/1.73m2) eGFR groups included small ‎numbers of patients (80 and 39, respectively). We also suspect that for the very low ‎eGFR group, the addition of a second mammary artery was indeed insignificant due ‎to the inherent short survival of this patient population.‎

The following figure depicts Kaplan Meier survival curves of each eGFR group according to the type of revascularization**.**

**S1 Figure 1: Kaplan-Meier survival curves for each estimated glomerular ‎filtration rate group: eGFR≤15, 15>eGFR≤30, 30<eGFR≤45, and eGFR>45 mL/min/1.73m2***
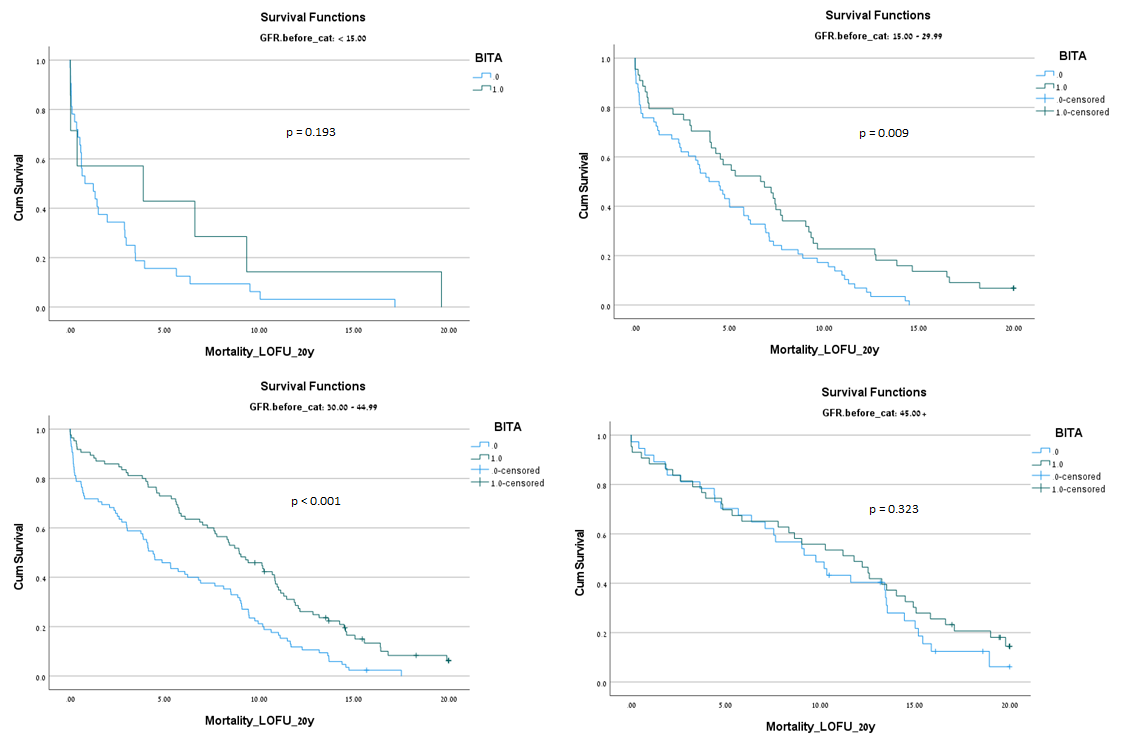
*

BITA: bilateral internal thoracic artery grafting, eGFR: estimated glomerular filtration rate. LOFU: last date of follow-up.

The p-values for each group appear on the appropriate graph.
