# Supplementary material for: Single versus bilateral internal thoracic artery grafting in patients with impaired renal function
Source: PLoS One. 2024 Feb 14;19(2):e0297194. doi: 10.1371/journal.pone.0297194 (PMC10866522; doi:10.1371/journal.pone.0297194)
Supplement: S2 Fig — (DOCX) [file pone.0297194.s003.docx]

**S1 Figure 2**

The below plot visually depicts differences between the two groups before and after matching. The numerical values are detailed in Table 1 of the article.



**S1 Figure 2: Standardized mean difference plot before and after matching**

CHF: congestive heart failure, COPD: chronic obstructive pulmonary disease, DM: diabetes mellitus, DM_EOD: diabetes mellitus with end-organ damage, IDDM: insulin dependent diabetes mellitus, EF: ejection fraction, eGFR: estimated glomerular filtration rate, IABP: intra-aortic balloon pump, IDDM: insulin dependent diabetes mellitus, MI: myocardial infraction, NOVS: number of vessels, OPCAB: off-pump coronary artery bypass, PCI: percutaneous coronary intervention, PVD: peripheral vascular disease, SVG: saphenous vein graft, PVD: peripheral vascular disease, GEA: gastroepiploic artery
