# Supplementary material for: Single versus bilateral internal thoracic artery grafting in patients with impaired renal function
Source: PLoS One. 2024 Feb 14;19(2):e0297194. doi: 10.1371/journal.pone.0297194 (PMC10866522; doi:10.1371/journal.pone.0297194)
Supplement: S1 Table — (DOCX) [file pone.0297194.s004.docx]

**S1 Table 1**

We performed a univariate analysis (Kaplan – Meier curves, log-rank test and Cox regression) to identify the patients who would benefit from BITA revascularization. We found that BITA revascularization was associated with better survival in patients **without** the following comorbidities: IDDM (p<0.001), EF<30 (p<0.001), IABP (p<0.001), critical condition (p<0.001), emergency operation (p<0.001), redo operation (p<0.001), PVD (p<0.001) and left main coronary involvement (p<0.001).

**S1 Table 1: Univariate Cox regression of additional factors that significantly affected 20-year survival of patients with chronic renal failure who underwent bilateral internal thoracic artery grafting**

| **HR (95%CI), p value** | **Characteristic** |
| --- | --- |
| HR=0.556 (0.445-0.695), p<0.001 | No IDDM |
| HR=0.571 (0.455-0.715), p<0.001 | No EF<30 |
| HR=0.53 (0.424-0.662), p<0.001 | No IABP |
| HR=0.523 (0.419-0.654), p<0.001 | No critical state |
| HR=0.524 (0.413-0.664), p<0.001 | No emergency surgery |
| HR=0.543 (0.438-0.674), p<0.001 | No redo operation |
| HR=0.457 (0.345-0.604), p<0.001 | No PVD |
| HR=0.539 (0.433-0.671), p<0.001 | No neurological damage |
| HR=0.523 (0.405-0.676), p<0.001 | No LM disease |

EF: ejection fraction, IDDM: insulin dependent diabetes mellitus, IABP: intra-aortic balloon pump, LM: left main artery disease, PVD: peripheral vascular disease
